# Geometry‐Controlled Assembly of Self‐Standing Nanorods With Undisturbed Plasmonics

**DOI:** 10.1002/advs.75726

**Published:** 2026-05-29

**Authors:** Yoel Negrin‐Montecelo, I. Brian Becerril‐Castro, Vladimir A. Baulin, Veronica Salgueiriño, Ramon A. Alvarez‐Puebla, Miguel A. Correa‐Duarte

**Affiliations:** ^1^ CINBIO Campus Universitario Lagoas Universidade De Vigo Vigo Spain; ^2^ Department of Physical and Inorganic Chemistry Universitat Rovira I Virgili Tarragona Spain; ^3^ Department of Chemical Engineering Universitat Rovira I Virgili Tarragona Spain; ^4^ Institució Catalana de Recerca i Estudis Avançats—ICREA Tarragona Spain; ^5^ Southern Galicia Institute of Health Research (IISGS), and Biomedical Research Networking Center for Mental Health (CIBERSAM) Pontevedra Spain

**Keywords:** geometry‐controlled assembly, plasmonic nanoparticles, SERS

## Abstract

The ability to control the spatial organization of nanoscale building blocks into well‐defined architectures remains a major challenge in materials science, as collective optical, electronic, magnetic, and catalytic properties often emerge from their precise arrangement. In plasmonic systems, coupling between localized surface plasmon resonances (LSPR) enables nanoscale light manipulation, yet current assembly strategies typically produce disordered architectures that limit practical applications. Here, we report a geometry‐controlled assembly approach that directs gold@silver core–shell nanorods into vertically aligned configurations on individual colloidal templates. By exploiting a size‐dependent “magic number” effect between nanorods and templates, we precisely control interparticle spacing and orientation, preserving the intrinsic optical response of individual nanorods while enabling collective mesoscale control. This strategy provides a general framework for assembling nanostructures with tunable optical properties, bridging colloidal dispersions and functional solid‐state architectures. As a proof of concept, we demonstrate highly reproducible surface‐enhanced Raman scattering (SERS) platforms with enhancement factors exceeding 10^6^ and excellent uniformity. Beyond SERS, this approach offers a versatile route for engineering plasmonic and hybrid materials for photonics, sensing, and nanoscale energy conversion, where geometry‐driven interactions determine functional performance.

## Graphical abstract

This study reports a geometry‐driven assembly strategy to vertically align Au@Ag core–shell nanorods on PS beads. This well‐defined architecture enables the preservation of the intrinsic optical response of individual nanorods. Thus, demonstrate a highly reproducible SERS enhancement with exceptional uniformity. This general strategy provides a bridge between colloidal dispersions and functional solid‐state architecture.

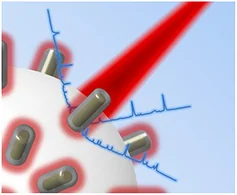

## 1 Introduction

The optical properties of plasmonic nanoparticles have been the subject of extensive research over the past four decades. When illuminated with light of appropriate wavelength, these nanoparticles generate strong electric fields at their surfaces due to localized surface plasmon resonances (LSPR). The LSPR characteristics can be finely tuned by adjusting parameters such as particle size, shape, composition, and the geometric arrangement of interacting or aggregated particles. For a given material, particle size has a relatively minor influence on the LSPR peak position, typically inducing redshifts of only a few tens of nanometers even with significant increases in colloidal diameter [1]. In contrast, shape plays a critical role, with variations in morphology leading to LSPR shifts spanning hundreds of nanometers for particles of comparable size [2]. Composition also profoundly affects the optical behavior of plasmonic materials, influencing both reactivity and stability. For instance, spherical nanoparticles of similar dimensions exhibit LSPR peaks ranging from 143 nm for aluminum to 555 nm for potassium, while commonly used materials such as silver and gold display peaks at 361 and 516 nm, respectively [3]. Furthermore, in binary alloys of gold and silver, the LSPR wavelength can be systematically modulated—redshifting when silver nanoparticles are coated with gold [4] or blueshifting when gold nanoparticles are coated with silver [5]. Additionally, particle aggregation invariably results in a severe redshift of the LSPR, accompanied by the formation of electromagnetic hotspots [6, 7]. This plasmon tunability has been exploited to produce controlled aggregates, though traditional methods often yield poorly defined structures. For example, random aggregation can be induced by increasing ionic strength through the addition of alkaline salts [8, 9] or by introducing hydrophobic organic molecules in aqueous colloidal solutions [10]. However, these approaches lead to uncontrolled aggregation, producing polydisperse clusters with highly variable sizes, shapes, and interparticle distances. Consequently, the resulting optical effects are neither reproducible nor predictable. To address these limitations, alternative strategies have been developed to precisely control aggregate size, morphology, and spatial arrangement. These methods primarily involve two approaches. The first relies on functionalizing nanoparticles with specific capping agents such as surfactants [11], nucleic acids [12], organic molecules [13], or polymers [14] to direct their assembly into ordered colloidal crystals [15]. The second approach employs larger colloidal particles, either plasmonic or optically inert, as templates to organize smaller nanoparticles into well‐defined structures on their surfaces [16, 17]. A common outcome of these methods is the generation of densely packed hotspots, which are advantageous for applications like ultrasensitive detection but may be undesirable when the intrinsic optical properties of the individual nanoparticles need to be preserved [7, 18, 19].

Herein, we design and synthesize a material in which gold@silver core–shell nanorods are assembled in an individual standing‐up configuration on a structured surface while preserving their intrinsic optical properties as observed in colloidal dispersion. This controlled organization is achieved by exploiting the geometric ratios between the nanorods and the substrate, ensuring optimal spacing and orientation. Specifically, we leverage a critical size‐dependent parameter to dictate the deposition geometry and packing density of the nanorods on the surface. This approach allows for precise control over interparticle interactions, minimizing undesired plasmonic coupling that could otherwise alter their optical response. Furthermore, we systematically evaluate the optical properties of these organized nanostructures, with particular emphasis on their performance in surface‐enhanced Raman scattering (SERS) spectroscopy [20, 21]. By correlating the geometric parameters of the system with SERS enhancement factors, we establish design principles for optimizing plasmonic substrates for analytical applications.

## 2 Results and Discussion

**FIGURE 1:**
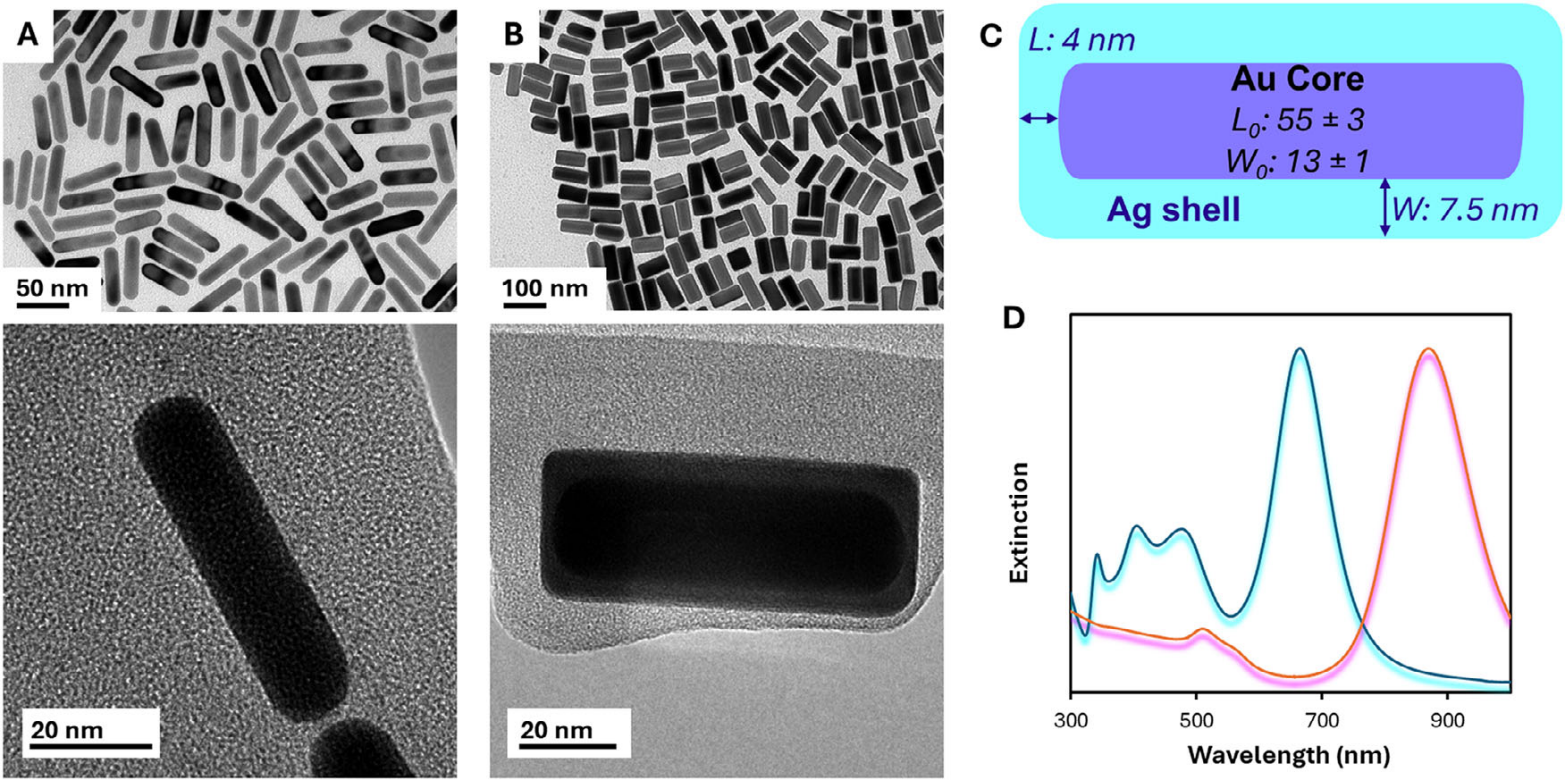
(A) TEM images of gold and (B) gold@silver nanorods. (C) Scheme showing the geometrical dimensions of the prepared materials. (D) Optical properties of the Au (pink) and Au@Ag (blue) nanorods with longitudinal LSPRs at 840 and 665 nm, respectively.

Figure 1 illustrates gold‐silver core–shell nanorods (Au@AgNRs) used as building blocks. Initially, gold nanorods (AuNRs) with an average length (L) of 55 nm and diameter (a) of 13 nm were synthesized, exhibiting a dominant longitudinal localized surface plasmon resonance (LSPR) peak at 840 nm due to their anisotropic morphology [22]. These AuNRs were then epitaxially overgrown with silver, resulting in squared core–shell nanorods with final dimensions of L = 71 nm and a = 28 nm [18]. The silver coating modifies both the external geometry and the internal electronic structure of the particles, leading to a blue shift of the longitudinal LSPR from 840 to 665 nm [5, 19, 23, 24]. Additional electrodynamic calculations performed for Au, Ag, and Au@Ag nanocuboids with identical external dimensions (Figure S1) show that the Au@Ag core–shell structure is not optically equivalent to the monometallic Ag analogue, but instead exhibits a distinct intermediate response arising from the combined effects of Ag‐shell growth and plasmonic interaction between the Au core and Ag shell.

To facilitate colloidal stability and subsequent assembly, the benzyldimethyldodecylammonium chloride (BDAC)‐stabilized Au@AgNRs were functionalized with an anionic polyelectrolyte (polystyrenesulfonate, PSS) coating, yielding a highly negative surface charge (ζ‐potential = −43.3 mV). Conversely, 280 nm polystyrene (PS) beads were modified with poly(allylamine hydrochloride) (PAH), imparting a strong positive charge (ζ = +31 mV), to enable electrostatic‐driven heteroassembly with the PSS‐coated nanorods.

**FIGURE 2:**
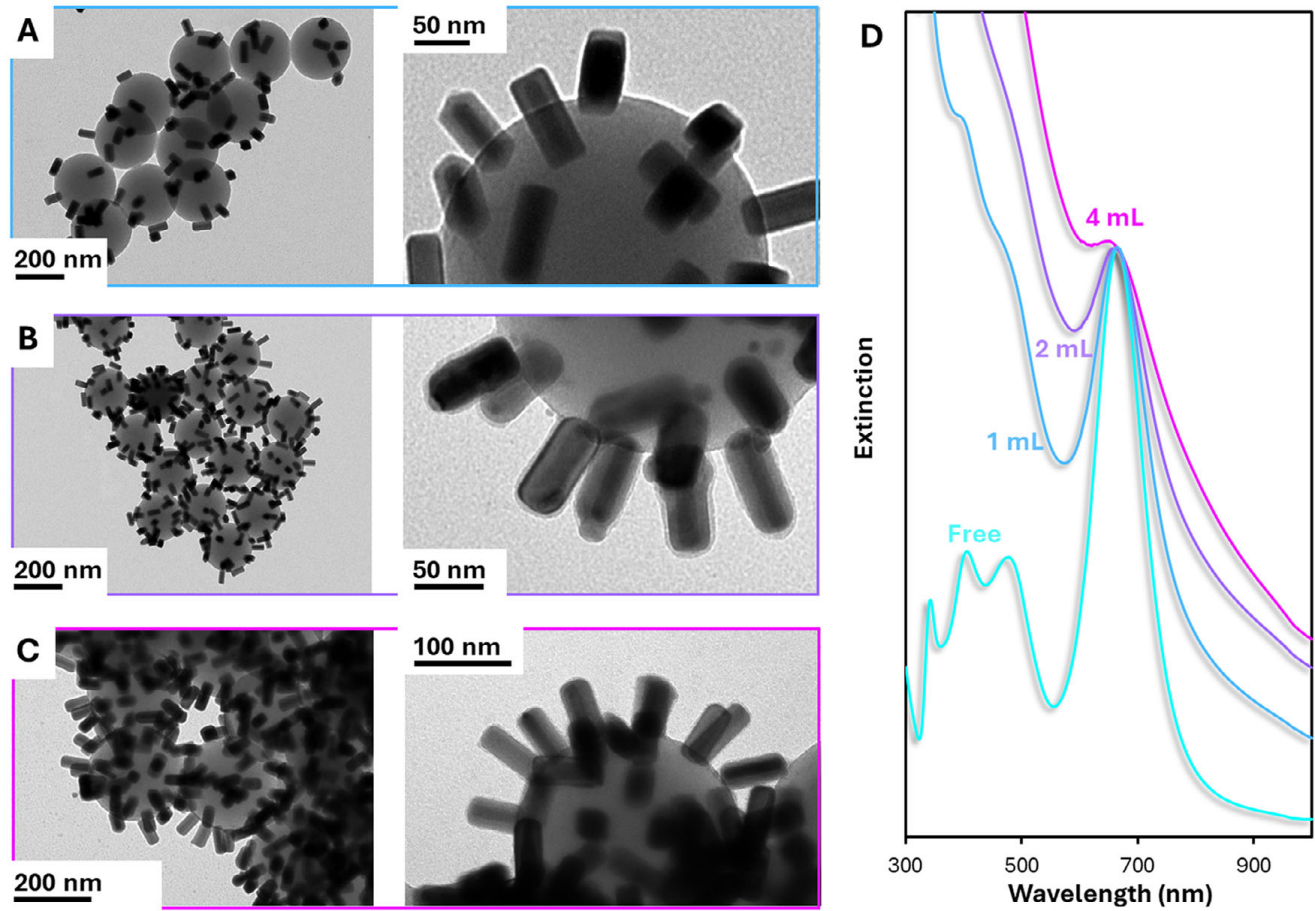
TEM images of different concentrations (A) 1 mL; (B) 2 mL; and (C) 4 mL of Au@Ag NR 1 mM adsorbed onto PS beads of 280 nm. (D) Optical properties of the obtained materials as compared with the free original Au@Ag NRs in solution.

The electrostatic assembly process was conducted using a constant concentration of PS beads (5 mL of 0.2 mg/mL suspension, ∼3.8 × 10^10^ beads) The nanorod concentration was precisely quantified through ICP‐OES measurements, which confirmed an Au/Ag molar ratio of 0.3 and a total metal concentration of 1 mM, corresponding to approximately 1.2 × 10^1^ ^5^ nanorods per liter based on the calculated metal atom content per nanorod (∼500,000 atoms/rod from geometric and compositional analysis). The assembly process involved dropwise addition of 1–4 mL nanorod suspension to the PS bead solution under controlled stirring conditions (3 h at room temperature). This resulted in the formation of hybrid nanostructures in which the nanorods adopted a predominantly standing‐up orientation on the bead surfaces, as evidenced by the TEM images in Figure 2. For the present system, TEM is particularly well suited to this analysis because it directly reveals the full projected hemisphere of each bead and, at sufficiently high magnification, resolves the nanorod disposition on the bead surface with high clarity, especially at the bead contour where standing‐up and lying‐down configurations can be distinguished most reliably. The maximum theoretical coverage was estimated by considering geometric constraints and electrostatic factors: each standing nanorod occupies a footprint of 784 nm^2^ (28 × 28 nm), while the available surface area of a 280 nm bead is approximately 246,300 nm^2^, yielding a theoretical maximum of 314 rods per bead under ideal close‐packing conditions. Experimental counts from TEM analysis were lower but remained highly consistent across the different nanorod concentrations. The number of nanorods per bead was estimated by counting the nanorods visible on the projected hemisphere of individual beads and extrapolating to the full particle assuming approximate hemispherical symmetry of the surface coverage. More than ten beads were analyzed for each condition. Using this approach, we obtained 9 ± 4 rods per bead (22.5 ± 10% coverage) for 1 mL addition, 18 ± 5 rods per bead (21.9 ± 6.3% coverage) for 2 mL, and 33 ± 3 rods per bead (20.6 ± 1.9% coverage) for 4 mL. The consistent adsorption efficiency of approximately 21% across all concentrations suggests that the assembly process is governed by balanced electrostatic interactions between the negatively charged PSS‐coated nanorods and the positively charged PAH‐functionalized beads, while steric hindrance from the polymer layers likely limits higher packing densities.

Detailed spectroscopic characterization of the hybrid nanostructures provided important insight into their plasmonic properties. As shown in Figure 2D, the longitudinal LSPR peak remained centered at 665 nm across all assembly conditions, with no appreciable red shift and only minimal broadening relative to the colloidal nanorods. This spectral stability indicates that strong longitudinal end‐to‐end coupling is effectively suppressed in the assembled structures. It also supports several key features of the system: (i) the predominantly standing‐up orientation of the nanorods is preserved over the investigated surface coverages, since extensive lying‐flat configurations or uncontrolled aggregation would be expected to induce more pronounced changes in the longitudinal response; (ii) the average inter‐rod distance remains large, consistent with the separation between particles. Using the measured rod loadings on the 280 nm beads and the total bead surface area, the average edge‐to‐edge separation at the bead surface can be estimated to be approximately 137, 89, and 58 nm for the 1, 2, and 4 mL conditions, respectively, and this separation increases further along the rod body because the nanorods stand radially on a curved surface. Thus, not reaching the narrow‐gap regime typically associated with strong plasmonic coupling and the most intense hot‐spot formation [25, 26, 27]; (iii) the local dielectric environment around each nanorod remains relatively homogeneous, with the surrounding aqueous medium providing the dominant contribution. Although some modifications are observed in the transverse spectral region, these are therefore more consistent with limited lateral or transverse interactions than with a strongly coupled regime [25, 26, 27]. Thus, the key result is not the complete absence of plasmonic interactions, but the preservation of a controlled coupling regime in which strong aggregation‐induced perturbations of the dominant longitudinal mode are avoided. The invariance of the longitudinal LSPR position with increasing nanorod loading further supports the precision and reproducibility of the assembly process, making these hybrid structures particularly attractive for applications requiring controlled plasmonic field enhancement and consistent optical response.

**FIGURE 3:**
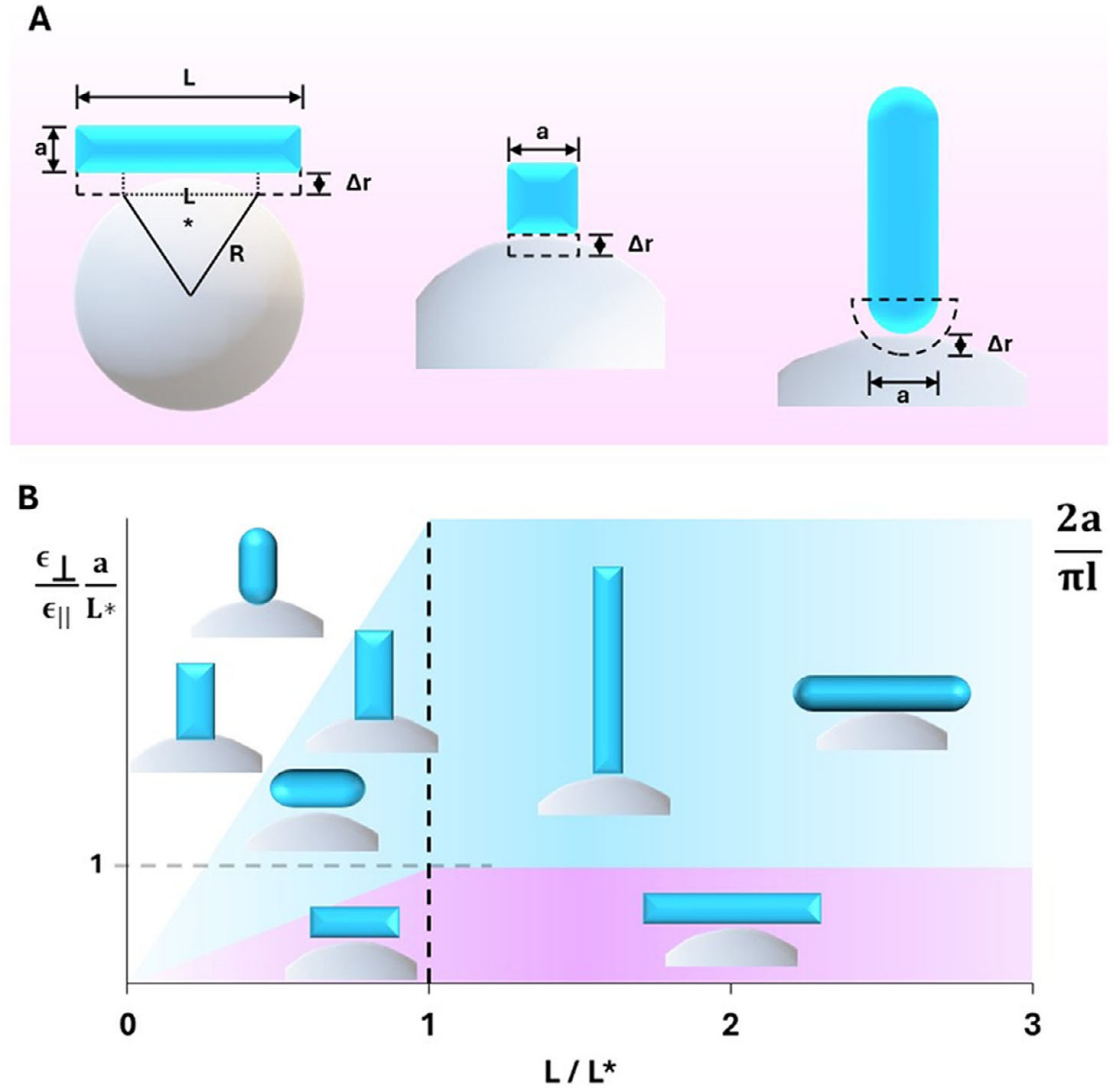
(A) Schematic representation of the interaction between an elongated nanoparticle of length L (exceeding the critical interaction length L*) and a spherical substrate of radius R. The left panel shows a frontal view and the center panel shows a side view of the nanoparticle in parallel orientation (∥); the right panel depicts the nanoparticle in perpendicular orientation (⊥). The interaction characteristics are determined by the tip geometry: for interaction ranges ∆𝑟 ≪ 𝑎, the contact area of a flat tip exceeds that of a hemispherical tip. (B) State diagram illustrating the orientation of an elongated nanoparticle adsorbed on a spherical substrate. The white region represents the parameter space favoring perpendicular orientation, while the colored region corresponds to parallel orientation. Pink indicates parallel orientation for nanoparticles with flat tips, whereas blue denotes the transition regime where nanoparticles with hemispherical tips adopt parallel orientation while those with flat tips remain perpendicular.

To explain this retention process of the NRs onto the beads, we developed a geometric model that analyzes nanoparticle orientation on spherical substrates. The model considers elongated nanoparticles of length *L* with a square cross‐section of side length a, attached to the surface of a sphere of radius *R*. Under the assumption that particle‐particle interactions are negligible for the NR concentration, the system reduces to a single‐particle interaction problem. Consequently, the analysis focuses on a single elongated nanoparticle that can adopt two distinct orientations relative to the spherical surface: parallel (∥) or perpendicular (⊥) (Figure 3). The perpendicular dimension of the nanoparticle is assumed to be significantly smaller than the sphere's radius (*a* ≪ *R*,), while the length (*L*) may vary, being either smaller or larger than (*R*).

The interaction between the elongated particle and the sphere is determined by geometrical considerations only, namely the curvatures of the tip and the sphere. If the nanoparticle is touching the sphere, the interaction is determined by a certain contact area around the contact point, which depends on the curvature and the geometry of the nanoparticle and the sphere, which is multiplied by the corresponding interaction parameter, ε_∥_ for parallel orientation and ε_⊥_ for perpendicular orientation. To calculate the contact area, we assume the interaction range Δ*r* around the particle in the form of the square well potential, such that they interact only with the parts of the surface of the sphere that are inside the interaction range within the Δ*r* distance from the particle. Curved geometry establishes a critical threshold for the maximum length of the cylinder, L*. Short particles *L* < L* are interacting across total length and it grows linearly with the length (adsorbed regime), and beyond this threshold *L* > L* the contact area does not increase further with longer length but stays constant (suspended regime), Figure 3A,

$$\;L^{*}=2R\sqrt{2\frac{\Delta r}{R}-{\left(\frac{\Delta r}{R}\right)}^{2}}$$

Thus, the geometry of the particles determines the contact area in two regimes: (i) parallel orientation: the interaction area between the particle and the sphere, *A*_∥_ =  *aL*, if *L* < L* and *A*_∥_ =  aL*, if *L* ≥ L* and (ii) perpendicular orientation: in case of flat tip *A*_⊥_ = *a* ^2^  and in case of a hemi‐spherical tip *A*_⊥_ = π*l* ^2^ , where *l* is the radius of the intersection of the interaction range and the sphere. It is given by

$$\;{\left(\frac{l}{a}\right)}^{2}=\frac{4\Delta r}{a}\;\frac{\left(1+\frac{\Delta r}{a}\right)}{{\left(1+\frac{2R}{a}\right)}^{2}}\left[{\left(1+\frac{4R}{a}\right)}^{2}-{\left(1+\frac{2\Delta r}{a}\right)}^{2}\right]$$

In this model, the orientation of the particle, whether in parallel or perpendicular orientation, is solely determined by the balance of its interactions in each orientation. If

$$\frac{\varepsilon_{⊥}A_{⊥}}{\varepsilon_{∥}A_{∥}}>1$$

the particle will be in parallel orientation, if this expression <1, it will be in perpendicular orientation.

The relationship between geometrical parameters can be illustrated using a state diagram, Figure 3B. The radius of the sphere *R* and interaction range Δr fix the threshold length L* and the parameter that controls the interaction is $\frac{\varepsilon_{⊥}a}{\varepsilon_{∥}L^{*}}$. Larger ε_∥_ and larger threshold length signifies dominance of the lateral contact over tip contact, while larger ε_⊥_ and larger tip size *a* favor contacts with the tip and perpendicular orientation. In the suspended regime, *L* > L*, the length does not play a role, and the orientation is controlled by the interplay between lateral and tip interactions. In case of a spherical tip, the boundary between the regimes lifts to 2*a*/π*l* making perpendicular orientation less favorable or even impractical.

**FIGURE 4:**
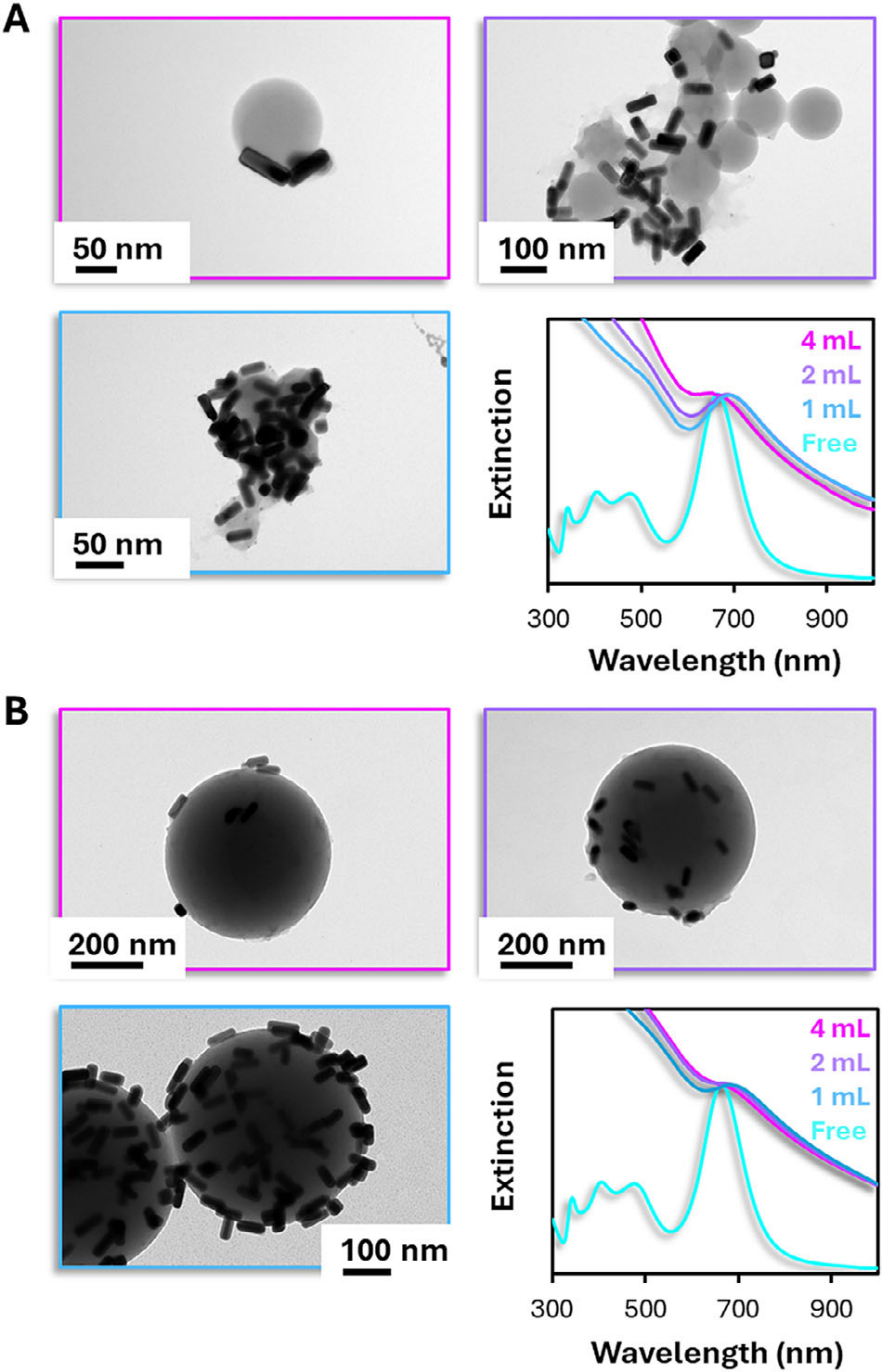
TEM images and LSPRs of different concentrations 1 mL, 2 mL, and 4 mL of Au@Ag NR 1 mM adsorbed onto PS beads of (A) 150 nm and (B) 450 nm.

To confirm this model the adsorption of the same concentration of rods was carried out onto beads of different diameters, 150 and 450 nm (Figure 4). Our comprehensive experimental study of nanorod assembly on spherical substrates reveals a precise curvature‐dependent orientation control, with three distinct regimes emerging from systematic variation of bead diameter. For the optimal 280 nm beads, we observe well‐defined standing‐up configurations characterized by an adsorption density of 22.5 ± 6.5 rods per bead, representing 21.3 ± 5.8% surface coverage. This intermediate size provides ideal geometric parameters with a curvature of κ ≈ 0.0071 nm^−^^1^ and available surface area of 0.246 µm^2^ per bead, creating conditions where the critical length ratio L/R ≈ 0.45 (for 63 nm rods on 140 nm radius beads) satisfies the model's condition for perpendicular orientation (ε⊥A⊥)/(ε∥A∥) = 1.8 ± 0.3 > 1. The optical signatures (Figure 2D) confirm this structural arrangement, showing a stable longitudinal LSPR peak at 665 nm with a narrow bandwidth (FWHM = 32 nm), indicating minimal inter‐rod coupling and consistent local electromagnetic environments.

In contrast, assembly on 150 nm beads (Figure 4A) produces disordered adsorption patterns due to excessive curvature (κ ≈ 0.013 nm^−^^1^) and limited surface area (0.071 µm^2^). These systems exhibit only 8 ± 15 rods per bead (7.5 ± 4.7% coverage) with <5% perpendicular alignment, resulting from both geometric frustration (L/R ≈ 0.84) and unfavorable energy ratios (ε⊥A⊥)/(ε∥A∥) = 0.3 ± 0.2. The optical response reflects this disorder through peak broadening (FWHM = 48 ± 5 nm) and redshift (695 nm), while reduced absorbance intensity (0.42 a.u.) indicates incomplete surface coverage. At the opposite extreme, 450 nm beads (κ ≈ 0.0044 nm^−^^1^) promote lying‐flat configurations with 93 ± 18 rods per bead (33.2 ± 7.6% coverage) and >90% parallel alignment, driven by favorable energy landscapes (ε⊥A⊥)/(ε∥A∥) = 0.6 ± 0.1 and optimal length ratios (L/R ≈ 0.28). These assemblies show characteristic optical signatures including a red‐shift (675 nm) from substrate coupling and higher absorbance (1.12 a.u.) from increased metal loading.

The complete dataset demonstrates remarkable agreement with our geometric model, showing <10% deviation across all measured parameters. The transition between orientation regimes occurs at critical length ratios, with L/R > 0.6 leading to disordered adsorption, 0.3 < L/R < 0.6 enabling standing‐up configurations, and L/R < 0.3 promoting lying‐flat orientations. Energy landscape analysis reveals the underlying physics, with calculated interaction ratios explaining orientation preferences (ε⊥/ε∥ ≈ 2.1 for 280 nm beads favoring perpendicular alignment versus 0.7 for 450 nm beads favoring parallel arrangements). These findings establish quantitative design rules for engineering plasmonic hybrids, where 280 nm beads are optimal for standing‐up configurations (200‐350 nm diameter range), 450 nm beads maximize metal loading, and intermediate systems offer tunability for specific applications. The precise correlation between structural characterization and optical measurements validates the model's predictive power while providing practical guidelines for applications ranging from surface‐enhanced spectroscopy (standing‐up configurations) to plasmon‐enhanced catalysis (lying‐flat arrangements).

**FIGURE 5:**
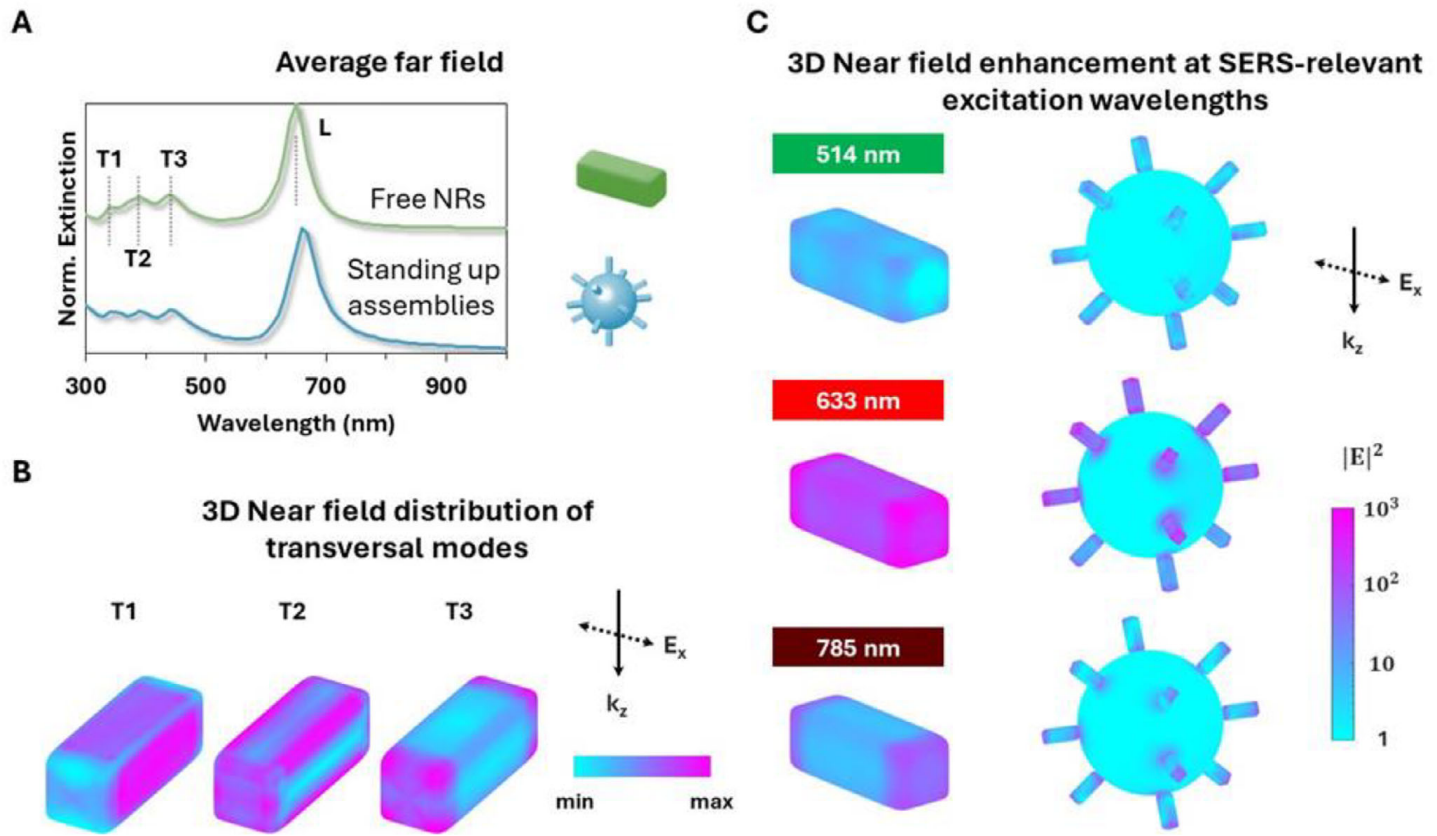
Simulated optical properties of Au@Ag NR and the standing up assembled materials. (A) Average far‐field extinction spectra comparing both structures, highlighting transverse (T1, T2, T3) and longitudinal (L) plasmon resonance modes of the Au@Ag NR. (B) 3D near‐field distribution (∣E∣^2^) of the Au@Ag NR's transverse plasmon modes (T1, T2, T3), illustrating electric field localization on faces, vertices, and corners. (C) Near‐field enhancement at SERS‐relevant excitation wavelengths.

The optical properties of Au@Ag NRs and their assembled standing up structures were computationally investigated to understand their plasmonic responses in both far‐field and near‐field regimes (Figure 5). The far‐field extinction spectra (Figure 5A) revealed three distinct transverse modes (T1, T2, T3) between 300 and 500 nm and a dominant longitudinal mode (L) near 650 nm for the Au@Ag NRs, consistent with previous reports on anisotropic silver nanostructures [28]. The assembled structures exhibited minimal spectral shifts (<5 nm) and slight broadening compared to isolated NRs, suggesting the polymer interlayers (PAH, PSS, BDAC) between the PS beads and NRs effectively maintained the dielectric environment while preventing detrimental plasmonic coupling. This observation contrasted sharply with the significant redshifts observed in strongly interacting NR systems, supporting the design principle of minimized inter‐rod interactions. Analysis of the 3D near‐field distributions (Figure 5B). showed characteristic spatial patterns for each transverse mode. The T1 mode displayed broadly distributed enhancement across the nanorod faces, while the T2 and T3 modes exhibited progressively more concentrated field localization at the vertices and corners respectively. This graduated field confinement from faces to edges reflected the increasing mode order and corresponding charge distribution complexity. The vertex‐ and corner‐localized enhancements were particularly notable as potential sites for extreme field concentration beneficial for surface‐enhanced applications. The wavelength‐dependent near‐field response (Figure 5C) was systematically evaluated at key excitation wavelengths (514, 633, 785 nm) relevant to SERS. The simulations demonstrated optimal enhancement at 633 nm for both isolated NRs and the assembled standing up structures, with field hotspots persisting at the terminal ends of the standing‐up NRs in the assembled system. This preservation of characteristic enhancement patterns confirmed that the controlled vertical orientation successfully prevented the near‐field coupling and random hotspot redistribution typical of aggregated NR systems. The most strongly enhanced NRs within the assembly were those aligned with the incident electric field polarization, mirroring the response expected for isolated rods.

**FIGURE 6:**
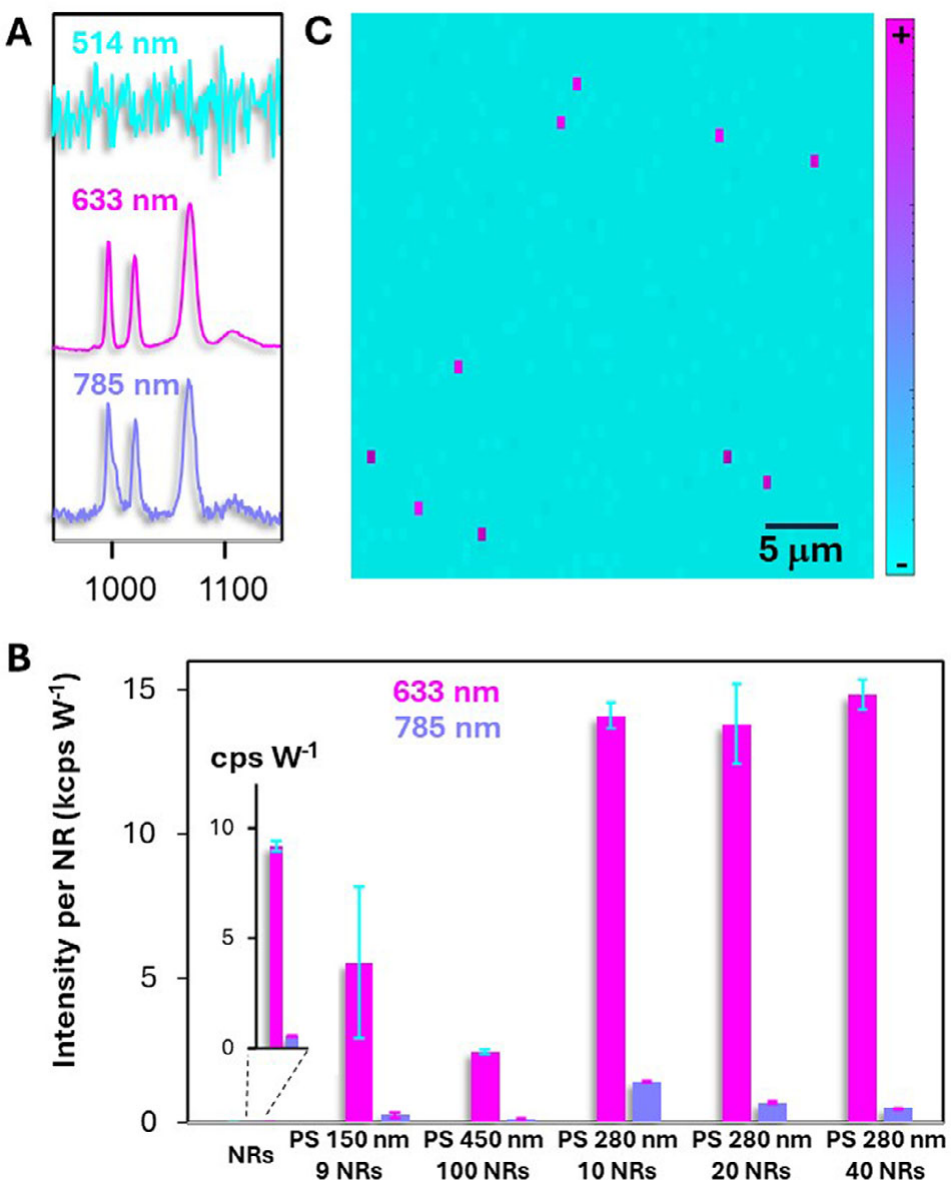
SERS characterization of benzenethiol adsorbed on Au@Ag nanorods and on assembled Au@Ag nanorod/PS‐bead structures. (A) Representative SERS spectra recorded with 514, 633, and 785 nm excitation. No detectable signal is observed at 514 nm, whereas 633 and 785 nm excitation produce the characteristic benzenethiol bands at 995, 1020, and 1070 cm^−1^. (B) Mean SERS intensity at 1070 cm^−1^ per nanorod for free Au@Ag nanorods and for the different assembled configurations under 633 and 785 nm excitation. Error bars represent the standard deviation. (C) Representative SERS intensity map at 1070 cm^−1^ of a single assembled structure spin‐coated on a silicon wafer. The map shows the spatial localization of the assembles and reproducibility of the SERS response. The color scale represents the relative Raman intensity.

The SERS response of benzenethiol (BT) at 10^−^^7^ m concentration was systematically evaluated across different plasmonic substrates using 514, 633, and 785 nm laser excitations (Figure 6A). As predicted by both theoretical LSPR calculations and experimental spectra, the 514 nm laser failed to generate detectable signals due to the absence of plasmonic resonance at this wavelength (Figure 6B). In contrast, excitation at 633 and 785 nm produced characteristic BT vibrational signatures: 1070 cm^−1^ (C─S stretching), 1020 cm^−1^ (ring breathing) and 995 cm^−1^ (ring deformation), with the 633 nm laser demonstrating 4.2‐fold greater enhancement than the 785 nm excitation (Figure 6B), consistent with its optimal spectral overlap with the longitudinal LSPR of the Au@Ag NRs.

For comparison across the different material configurations, the SERS intensities shown in Figure 6B were normalized to the acquisition time, the laser power at the sample, and the estimated number of probed nanorods. This normalization was introduced to provide a practical per‐particle comparison across the different experimental configurations. For free nanorods in colloidal suspension, the number of contributing particles was estimated from the nanorod concentration and the illuminated sampling volume defined by the macrolens geometry. In contrast, the assembled materials were analyzed after immobilization on silicon wafers under microscopic conditions, where diluted suspensions were spin‐coated to generate isolated bead events for individual Raman mapping [29, 30]. Because colloidal suspensions and solid‐supported assemblies do not constitute strictly identical measurement geometries, this comparison should not be interpreted as a fully substrate‐independent measure of intrinsic SERS superiority. Rather, it should be regarded as a normalized reference that enables a practical comparison of the signal generated per probed nanorod, in line with current recommendations for quantitative SERS analysis across different substrate formats [31, 32]. Within this framework, quantitative analysis of the 1070 cm^−1^ benzenethiol band revealed clear differences among the tested architectures. The 280 nm bead assemblies displayed the highest normalized SERS intensity together with the best reproducibility, whereas the 150 nm systems showed broader signal dispersion, consistent with their greater structural heterogeneity. The 450 nm systems exhibited lower intensities but narrower distributions. Overall, these results indicate that the standing‐up architecture provides the most favorable balance between signal enhancement and reproducibility under our experimental conditions.

## 3 Conclusions

In summary, here we demonstrate a geometry‐directed assembly strategy that enables the precise organization of anisotropic plasmonic nanoparticles into well‐defined, self‐standing configurations while maintaining their intrinsic optical properties. By establishing a quantitative relationship between nanoparticle dimensions, substrate curvature, and interaction energetics, we achieve controlled vertical alignment of Au@Ag core–shell nanorods on spherical templates. Systematic characterization reveals that 280 nm diameter templates provide optimal geometric matching, facilitating nanoparticle orientation that minimizes near‐field coupling while preserving individual plasmonic responses. This is evidenced by the invariant longitudinal LSPR peak position across varying surface coverages, confirming the absence of significant interparticle interactions. The assembled architectures exhibit exceptional performance as SERS substrates, delivering 1500‐fold signal enhancement relative to dispersed nanorods while maintaining high reproducibility (<10% RSD). This enhancement stems from the combined effects of accessible electromagnetic hotspots at nanorod termini and the suppression of inhomogeneous broadening through controlled spacing. The developed geometric model successfully predicts orientation behavior across different template sizes, establishing general design principles for nanoparticle assembly. These findings advance our understanding of structure–property relationships in plasmonic nanomaterials and provide a robust framework for engineering functional optical materials with tailored electromagnetic responses. From a practical standpoint, the present methodology is readily scalable at the colloidal level, since nanorod synthesis, bead functionalization, and electrostatic assembly are all solution‐based processes. The main limitations for device fabrication are therefore not associated with the synthesis itself, but rather with downstream integration, including control over template monodispersity, deposition density, long‐range ordering, and transfer of the hybrid objects onto robust macroscopic supports. Accordingly, the present strategy is particularly attractive for particulate coatings and ensemble optical/SERS substrates, whereas deterministic large‐area device fabrication will require additional processing approaches. More broadly, the methodology presented here may be extended to other anisotropic nanoparticle systems, opening new opportunities for the development of advanced plasmonic platforms for sensing, spectroscopy, and nanophotonic applications.

## Conflicts of Interest

The authors declare no conflicts of interest.

## Supporting information




**Supporting File**: advs75726‐sup‐0001‐SuppMat.docx.

## Data Availability

The data that support the findings of this study are available from the corresponding author upon reasonable request.
