# Supplementary material for: Geometry‐Controlled Assembly of Self‐Standing Nanorods With Undisturbed Plasmonics
Source: Adv Sci (Weinh). 2026 May 29:e75726. Online ahead of print. doi: 10.1002/advs.75726 (PMC13335839; doi:10.1002/advs.75726)
Supplement: Supplementary file 1 — Supporting File: advs75726‐sup‐0001‐SuppMat.docx. [file ADVS-9999-e75726-s001.docx]

Supporting Information

Geometry-Controlled Assembly of Self-Standing Nanorods with Undisturbed Plasmonics

Yoel Negrin-Montecelo,^[a],[b]^ I. Brian Becerril-Castro,^[b]^ Vladimir A. Baulin,^[c]^ Veronica Salgueirino,^[a],*^ Ramon A. Alvarez-Puebla,^[b],[d],^* Miguel A. Correa-Duarte^[a],*^

[a] Y. Negrin-Montecelo, V. Salgueirino, M. A. Correa-Duarte

CINBIO, Universidade de Vigo, Campus Universitario Lagoas, 36310, Vigo, Spain
E-mail: vsalgue@uvigo.gal, macorrea@uvigo.gal

[b] Y. Negrin-Montecelo, I. B. Becerril-Castro, R. A. Alvarez-Puebla

Department of Physical and Inorganic Chemistry, Universitat Rovira I Virgili, Marcel.lí Domingo 2-4-6, 43007, Tarragona, Spain
E-mail: ramon.alvarez@urv.cat

[c] V.Baulin

Department of Chemical Engineering, Universitat Rovira I Virgili, Marcel.lí Domingo s/n, 43007, Tarragona, Spain

[d] R. A. Alvarez-Puebla

Institució Catalana de Recerca i Estudis Avançats – ICREA, 08010, Tarragona, Spain

[e] M. A. Correa-Duarte

Southern Galicia Institute of Health Research (IISGS), and Biomedical Research Networking Center for Mental Health (CIBERSAM), Spain

Experimental Procedures

Materials

All chemicals were used as received without further purification. Tetrachloroauric acid trihydrate (HAuCl₄·3H₂O) and silver nitrate (AgNO₃) served as gold and silver precursors, respectively. Surfactants included hexadecyltrimethylammonium bromide (CTAB, 96%) and benzyldimethyldodecylammonium chloride (BDAC, 99%). Sodium borohydride (NaBH₄, 99%) and L-ascorbic acid (AA, 99%) were used as reducing agents. For surface functionalization, we employed poly(allylamine hydrochloride) (PAH, Mₙ ≈ 17,500) and poly(styrenesulfonate) (PSS, Mₙ ≈ 70,000). Additional chemicals included sodium citrate, sodium chloride, sodium formate, formic acid (99%), and hydrochloric acid (37%). Spherical polystyrene particles (PS) were purchased from Ikerlat Polymers. All aqueous solutions were prepared using Milli-Q water (resistivity ≥ 18.2 MΩ·cm).

Synthesis of the nanorods and PSS coating

Gold nanorods (AuNRs) with a longitudinal surface plasmon resonance (LSPR) band centered at 840 nm, an average length of 55 ± 3 nm, and a width of 13 ± 1 nm were synthesized using a modified version of the seed-mediated growth protocol reported by Scarabelli et al.[1] These AuNRs served as templates for the epitaxial growth of a silver shell, yielding Ag@Au core-shell nanorods (Ag@AuNRs) with a blue-shifted LSPR peak at 665 nm, reflecting the plasmonic hybridization between the gold core and silver shell. The final dimensions of the Ag@AuNRs were 71 ± 5 nm in length and 28 ± 2 nm in width, as determined by TEM analysis, following a previously established procedure.[2] Inductively coupled plasma optical emission spectroscopy (ICP-OES) confirmed an Au/Ag molar ratio of 0.3, indicating a substantial silver overgrowth. The as-synthesized Ag@AuNRs, stabilized by benzyldimethyldodecylammonium chloride (BDAC), initially exhibited a positive surface charge. To enable later electrostatic assembly with functionalized polystyrene (PS) beads, the nanorods were coated with a layer of the anionic polyelectrolyte poly(styrenesulfonate) (PSS). For this purpose, a PSS solution (2 mg/mL in 6 mM NaCl, pH 5.0) was prepared, and 20 mL of the NR dispersion was added dropwise to 40 mL of the PSS solution under continuous stirring (180 min, room temperature). Unbound PSS was removed via three centrifugation-redispersion cycles (6000 rpm, 20 min each), and the purified PSS-coated Ag@AuNRs were redispersed in 20 mL of Milli-Q water, yielding a stable colloidal suspension with a highly negative ζ-potential.

Synthesis and functionalization of PS beads

To enable electrostatic assembly with PSS-coated Ag@Au nanorods (Ag@AuNRs), polystyrene (PS) beads of varying diameters (150 nm, 280 nm, and 450 nm) were functionalized with poly(allylamine hydrochloride) (PAH) by first preparing a PAH solution (1 mg/mL in 0.5 M NaCl, pH 5.0) for optimal polyelectrolyte adsorption, then adding 12.5 mL, 22.5 mL, and 37.5 mL of this solution to 5 mL of 150 nm, 280 nm, and 450 nm PS bead dispersions (1 mg/mL), respectively, followed by 30 min of stirring at room temperature to ensure uniform PAH adsorption, after which the functionalized beads were purified through three centrifugation-redispersion cycles (8000 rpm, 30 min each) and finally redispersed in 25 mL of Milli-Q water to yield stable, positively charged PS-PAH colloids ready for assembly with PSS-coated Ag@AuNRs.

Assembly of Ag@AuNRs onto PS beads

For the electrostatic assembly process, varying volumes (1, 2, or 4 mL) of Ag@AuNRs@PSS solution (1 mM metal concentration) were added dropwise to 5 mL of each PAH-functionalized PS bead solution (0.2 mg/mL), with the mixtures then stirred at room temperature for 3 hours to facilitate binding, followed by three purification cycles via centrifugation and redispersion in water (3500 rpm, 30 min each cycle), ultimately yielding stable assemblies that were finally redispersed in 5 mL of water for further use.

Bead number estimation: assuming perfect spheres with density of 1.05 g/cm³, each 280 nm bead weighs ~1.13×10⁻¹⁴ g, making 0.2 mg/mL equivalent to ~1.77×10¹³ beads/L, thus 5 mL contains ~8.85×10¹⁰ beads; however, accounting for the typical 40-50% solid content in commercial PS latex suspensions (as these are surfactant-stabilized), the actual number is roughly half this value. For the different sizes used (150, 280, and 450 nm), the bead count scales inversely with volume (∼diameter): 150 nm beads would number ∼2.7×10¹¹, while 450 nm beads would be ∼3.3×10¹⁰ in the same 5 mL of 0.2 mg/mL solution.

Nanorod number estimation: To estimate the number of Ag@AuNRs added during assembly, we calculate that a 1 mM metal solution contains approximately 1.2×10¹⁵ nanorods/L (based on ~500,000 metal atoms per rod from their dimensions of 63×26 nm and Au/Ag ratio of 0.3), meaning 1-4 mL additions deliver 1.2 - 4.8×10¹² nanorods to 5 mL of 280 nm PS beads (0.2 mg/mL, ~3×10¹⁰ beads), resulting in roughly 40-160 rods per bead at full adsorption - though actual coverage may vary due to packing efficiency (theoretical maximum ~150 rods/bead) and potential aggregation effects during the electrostatic binding process.

Metal Nanoparticle Boundary Element Method

We use Boundary Element Method (BEM) approach,[3] and its implementation (MNPBEM17 toolbox[4] to describe the far and near field of the Au@Ag core-shell nanorods, and the composite nanoparticles. Dielectric constants employed for gold and silver are from Johnson & Christy.[5] and Palik’s Handbook[6] respectively. For the polystyrene sphere we use a refractive index n=1. 5875 and 1.33 for the media (water). The separation between rods and the central sphere was set to 5 nm. Every full rod was modelled as one gold rod embedded inside a silver superellipsoid[7] with ϵ_1_=ϵ_2_=0.2. Since our ensemble is symmetric with respect the x and y axis, MNPBEM17 code allowed us to simulate only one quarter of the full ensemble as shown in figure M0, the full solution was then obtained by symmetry operations inside the toolbox.

Characterization

Transmission electron microscopy (TEM) characterization was performed using a JEOL JEM 1010 instrument operating at 100 kV. Samples were prepared by drop-casting diluted colloidal dispersions onto carbon-coated copper grids followed by drying at room temperature. Optical properties were analyzed using a Cary 8454 UV-Vis-NIR spectrophotometer with spectra collected in 1 cm path length quartz cuvettes. For elemental quantification, samples were digested with hydrofluoric acid and analyzed by inductively coupled plasma optical emission spectrometry (ICP-OES) using a PerkinElmer Optima 4300 system. Colloidal stability was characterized by photon correlation spectroscopy (PCS) using a Malvern Zetasizer Nano ZS instrument.

SERS spectra were recorded in backscattering geometry using a Renishaw inVia Reflex Raman system equipped with a 2D-CCD detector and a Leica confocal microscope. Excitation was carried out with 514, 633, and 785 nm laser lines. For microscopic measurements on immobilized particles, spectra were acquired with an integration time of 1 s using a 50× long-working-distance objective, which provided a spatial resolution of approximately 1 μm. The laser power at the sample was 0.4 mW for 514, 016 mW for 633 nm excitation, and 1 mW for 785 nm excitation. For measurements in colloidal suspension, a macrolens with a focal length of 16 mm was used, providing an illuminated cylindrical volume of approximately 1 cm in length and 3 μm in diameter. In this configuration, the laser power at the sample was 16 mW for 633 nm excitation and 200 mW for 785 nm excitation.

All nanoparticles, either free or assembled, were additionally cleaned by four centrifugation steps (7000 rpm, 10 min) and redispersed in ethanol to reach a final metal concentration of 10^-3^ M. Each suspension was then incubated with benzenethiol to a final concentration of 10^-7^ M for 1 h before measurement or substrate deposition. For the colloidal SERS measurements, the suspension used for Raman analysis contained 10^-4^ M in total metal. The number of probed free nanorods was estimated from the nanorod concentration and the illuminated sampling volume. From ICP quantification, 1 mM in total metal corresponds to approximately 1.2 × 10¹² nanorods mL^-1^; therefore, at 10^-4^ M in metal the nanorod concentration was estimated as 1.2 × 10^11^ nanorods mL^-1^. Using the macrolens geometry, the illuminated volume was approximated as a cylinder of 1 cm length and 3 μm diameter, yielding approximately 7.1 × 10^-8^ mL and therefore about 8.5 × 10^3^ nanorods contributing to each colloidal spectrum. To obtain low particle densities for immobilized measurements (approximately <1 particle μm^-2^), 10 μL of a diluted nanoparticle suspension (10^-6^ M in metal) was spin-coated onto silicon wafers (0.5 × 0.5 cm) using the following program: first ramp, 500 rpm for 10 s; second ramp, 3000 rpm for 30 s; acceleration rate, 500 rpm s^-1^ for both ramps. This procedure yielded isolated bead events suitable for single-particle SERS mapping and analysis, following established single-particle confinement / isolated-event strategies on solid supports [8-11]. For quantitative comparison, the SERS signal was evaluated from the intensity of the benzenethiol band at 1070 cm^-1^. The intensities shown in Figure 6B were normalized to acquisition time, laser power at the sample, and the estimated number of probed nanorods.

TEM counting procedure for nanorods per bead

TEM was used to determine nanorod orientation and loading because it directly shows the full projected hemisphere of each bead and resolves the nanorod disposition most clearly at the bead contour. For each condition, the nanorods visible on the projected hemisphere of individual beads were counted manually and extrapolated to the full particle assuming approximate hemispherical symmetry of the surface coverage. More than ten beads were analyzed per sample, and the reported values are given as mean ± standard deviation.

The number of Au@Ag nanorods adsorbed on each PS bead was estimated from TEM micrographs. Since TEM provides a projected two-dimensional view, nanorod orientation was assessed mainly from the bead-contour regions, where standing-up and lying-down configurations can be distinguished most clearly. For particle counting, the nanorods visible on the projected hemisphere of each bead were counted manually, and the total number per bead was estimated by extrapolation assuming approximate hemispherical symmetry of the surface coverage ($N_{\text{total}}\approx2N_{\text{visible}}$). More than ten beads were analysed for each condition, and the reported values correspond to mean ± standard deviation. Surface coverage was calculated by comparing the experimental average with the theoretical maximum of 314 standing nanorods per 280 nm bead.

The average lateral separation between neighboring nanorods on 280 nm beads was estimated from the total bead surface area divided by the mean number of adsorbed nanorods per bead, assuming approximately homogeneous surface distribution. From the resulting mean area per nanorod, the average center-to-center distance was approximated as $d\approx\sqrt{A/N}$, and the corresponding edge-to-edge separation as $g\approx d-28$nm. This yielded mean gap estimates of approximately 137, 89, and 58 nm for the 1, 2, and 4 mL loading conditions, respectively.

References

[1] L. Scarabelli, A. Sánchez-Iglesias, J. Pérez-Juste, and L. M. Liz-Marzán, "A “Tips and Tricks” Practical Guide to the Synthesis of Gold Nanorods," *The Journal of Physical Chemistry Letters,* vol. 6, pp. 4270-4279, 2015-11-05 2015.

[2] S. Gómez-Graña, B. Goris, T. Altantzis, C. Fernández-López, E. Carbó-Argibay, A. Guerrero-Martínez*, et al.*, "Au@Ag Nanoparticles: Halides Stabilize {100} Facets," *The Journal of Physical Chemistry Letters,* vol. 4, pp. 2209-2216, 2013/07/03 2013.

[3] F. J. García de Abajo and A. Howie, "Retarded field calculation of electron energy loss in inhomogeneous dielectrics," *Physical Review B,* vol. 65, p. 115418, 03/08/ 2002.

[4] U. Hohenester and A. Trügler, "MNPBEM – A Matlab toolbox for the simulation of plasmonic nanoparticles," *Computer Physics Communications,* vol. 183, pp. 370-381, 2012-02-01 2012.

[5] P. B. Johnson and R. W. Christy, "Optical Constants of the Noble Metals," *Physical Review B,* vol. 6, pp. 4370-4379, 12/15/ 1972.

[6] D. W. Lynch and W. R. Hunter, "- Comments on the Optical Constants of Metals and an Introduction to the Data for Several Metals," in *Handbook of Optical Constants of Solids*, E. D. Palik, Ed., ed Burlington: Academic Press, 1997, pp. 275-367.

[7] M. B. Cortie, F. Liu, M. D. Arnold, and Y. Niidome, "Multimode Resonances in Silver Nanocuboids," *Langmuir,* vol. 28, pp. 9103-9112, 2012-06-19 2012.

[8] A. Mercedi, F. Cardoni, F. Toffanello, J. Reguera, M. Meneghetti, and L. Litti, "Reliable Methodology for Measuring SERS Enhancement Factor on Colloidal and Solid Substrates: A Practical Guide," *Journal of Raman Spectroscopy,* vol. 56, pp. 835-849, 2025.

[9] S. E. J. Bell, G. Charron, E. Cortés, J. Kneipp, M. L. de la Chapelle, J. Langer*, et al.*, "Towards Reliable and Quantitative Surface-Enhanced Raman Scattering (SERS): From Key Parameters to Good Analytical Practice," *Angewandte Chemie International Edition,* vol. 59, pp. 5454-5462, 2020.

[10] M. Liebel, I. Calderon, N. Pazos-Perez, N. F. van Hulst, and R. A. Alvarez-Puebla, "Widefield SERS for High-Throughput Nanoparticle Screening," *Angewandte Chemie International Edition,* vol. 61, p. e202200072, 2022.

[11] M. Blanco-Formoso, A. Sousa-Castillo, X. Xiao, A. Mariño-Lopez, M. Turino, N. Pazos-Perez*, et al.*, "Boosting the analytical properties of gold nanostars by single particle confinement into yolk porous silica shells," *Nanoscale,* vol. 11, pp. 21872-21879, 2019.


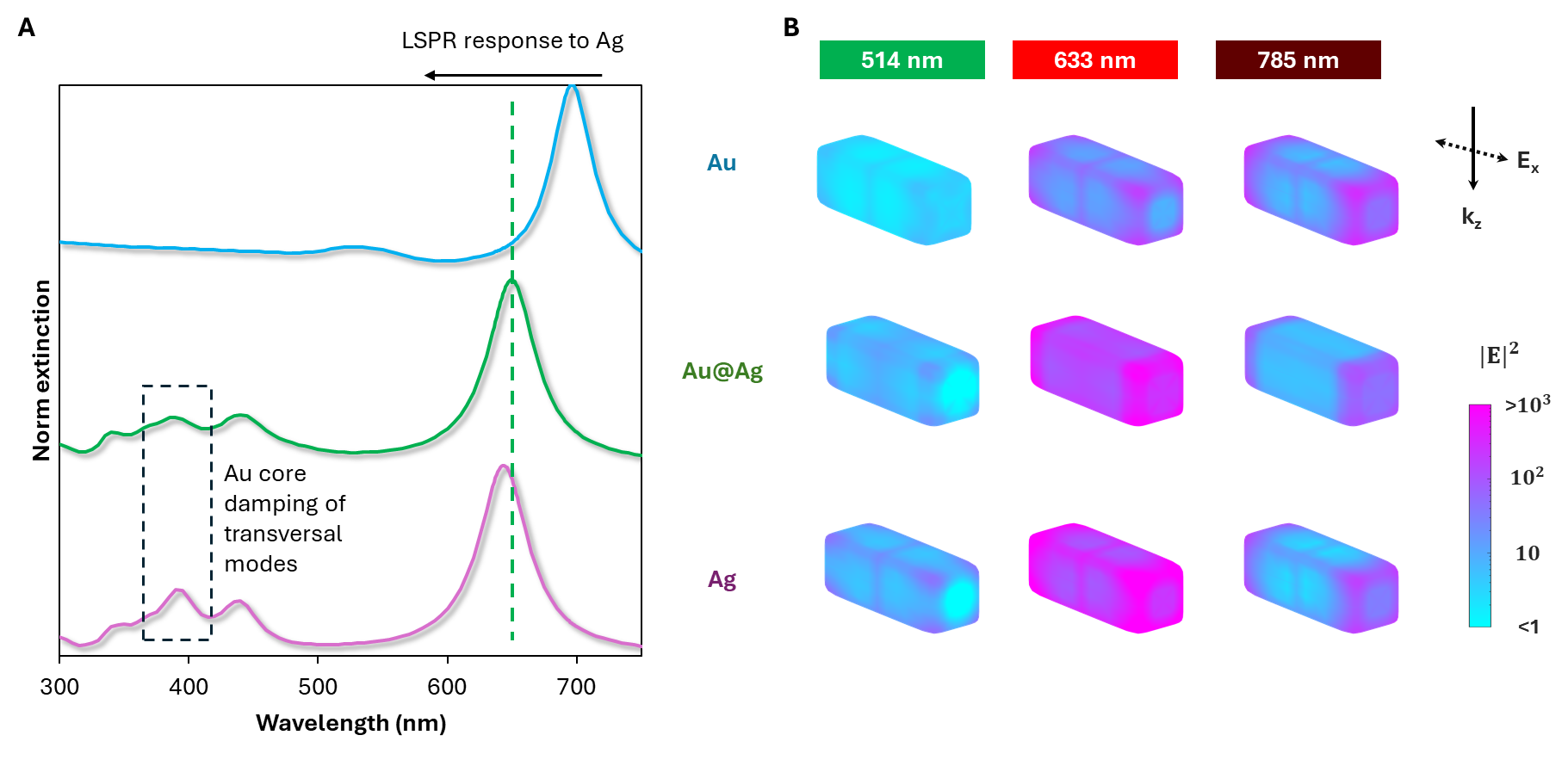


**Figure S1.** Electrodynamic calculations for Au, Au@Ag, and Ag nanorods with identical external dimensions. **(A)** Extinction spectra. The dashed vertical line marks the longitudinal LSPR position of the Au@Ag nanorod, and the dashed box highlights the transverse-mode region. The Au@Ag nanorod shows an intermediate spectral response between the Au and Ag limits, with measurable differences relative to the monometallic Ag nanorod. **(B)** Near-field intensity distributions $\left( \mid E\mid^{2} \right)$ at 514, 633, and 785 nm. While the field localization of Au@Ag and Ag nanorods is similar, their far-field optical responses are not identical.
